# From “people also ask” to clarifying questions for conversational search using parameter-efficient fine-tuning and prompt engineering

**DOI:** 10.1007/s00799-026-00445-z

**Published:** 2026-06-23

**Authors:** Isin Su Ecevit, Navdeep Singh Bedi, Ivan Sekulic, Fabio Crestani

**Affiliations:** Faculty of Informatics, Universitá della Svizzera Italiana (USI), Via Giuseppe Buffi 13, Lugano, CH-6900 Switzerland

**Keywords:** Conversational search, User intent clarification, Large language models, People also ask questions, Fine-tuning, Prompt-engineering

## Abstract

Conversational search systems enable natural, coherent dialogues between users and search systems to satisfy the user information need. The best of such systems improve user experience by asking clarifying questions (CQs) to resolve ambiguity in user queries. However, generating CQs that are both relevant and conversationally appropriate remains a significant challenge. To this end, we use Google’s “People Also Ask” (PAA) feature, grounding our questions in real-life user search behavior. We identify the most suitable methodology for this task by conducting an extensive comparative analysis of two methods: Parameter-Efficient Fine-Tuning (PEFT) and prompt engineering, and evaluate these using three state-of-the-art, lightweight language models. Furthermore, we introduce two new, human-annotated test sets derived from various conversational search datasets. Our results demonstrate that PEFT consistently and significantly outperforms all prompt engineering approaches across all models and test sets, and is statistically comparable to traditional full fine-tuning. These findings suggest that for the PAA-to-CQ task, investing in a high-quality dataset for efficient fine-tuning is a more reliable path to achieving high-quality, stylistically consistent outputs than relying on in-context learning alone.

## Introduction

Conversational information retrieval (CIR), or conversational search, is an area focused on enabling natural, coherent dialogues between users and search systems to satisfy the user information need. In contrast to traditional keyword-based search, CIR uses advanced natural language processing (NLP) and large language models (LLMs) to dynamically understand and respond to user needs [1]. CIR systems are capable of maintaining context over multi-turn interactions, clarifying user ambiguity, reformulating queries, and generating informative responses that are more than just a list of hyperlinks, which enhances user experience. CIR features mixed-initiative interactions, where both the user and the system can take the initiative to refine the information need across multiple turns [2, 3] collaboratively, in order for the system to build a better understanding of the user’s needs over multiple turns.

One of the main challenges for a CIR system is to accurately interpret ambiguous or under-specified user queries. Since user queries are often short and lack detail, some CIR systems use clarifying questions (CQs) to reduce the gap between user intent and query [4]. CQs have been shown to improve search performance, but their effectiveness is shaped by how they are phrased and how users respond [5]. Recent work has shown that poorly formulated CQs can lead to user frustration [6], which highlights the importance of creating high-quality CQs, as bad quality can negatively impact the search process [7].

There are two main methods for creating CQs. The first approach involves *selecting* an appropriate question from a predefined pool of questions, whereas the second approach focuses on *generating* the question. Although these two techniques are effective, they often lack a strong grounding in real-time user search behavior. Predefined question pools are often static and can quickly become outdated, failing to reflect emerging topics or the different ways users phrase their queries [8]. On the other hand, while pure LLM-based CQ generation is more flexible, it can sometimes produce questions that are less precise or contextually irrelevant, as they are not grounded in actual user search behavior.

**Fig. 1:**
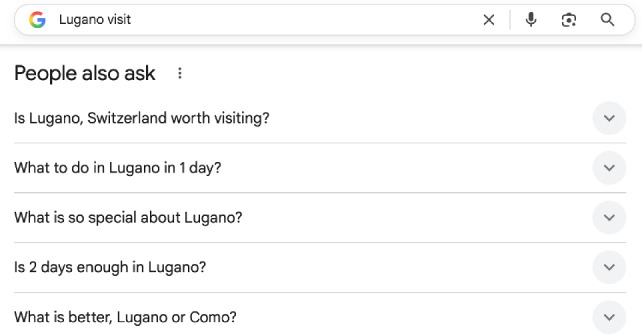
An example of Google’s People Also Ask feature for the given query

In this study, we extend our original work reported in [8], concerning the use of Google’s “People Also Ask” (PAA) algorithm to get relevant follow-up questions to generate clarifying questions related to a particular user query. PAAs are questions that are related to the search terms used in the query, and may be helpful as a next step to satisfy the user’s information need^1^. They are questions that people commonly search for in the topic of the current search. An example for the PAA feature can be seen in Figure 1. For a query such as *“Lugano visit”*, some related questions are *“Is Lugano, Switzerland worth visiting?”*, *“What to do in Lugano in 1 day?”*, and *“What is so special about Lugano?”*. When chosen, the system provides the user with a piece of text with a short answer to the question asked, which is called a “featured snippet”^2^. This way, the search experience is improved and the users are more likely to have their information need satisfied.

Using PAA for CQ generation has several important advantages that help mitigate the shortcomings of the current CQ studies. First, PAA questions are directly derived from real user search patterns, ensuring they are accurate, up-to-date, and closely aligned with what users are actively seeking to know. This provides real-time context that helps in understanding the user’s query more accurately. Unlike static, predefined question banks which can quickly become outdated, PAA questions are constantly updated based on current trends and popular queries, allowing for adaptable and flexible content generation. Furthermore, using PAA offers distinct advantages over relying solely on LLM-generated questions. While LLMs can be insightful, they may sometimes produce less relevant or contextually unaware queries. PAA questions, being grounded in actual user behavior, are context-aware of current interests and often cover various facets of a topic, offering a range of specific and appropriate clarifications. This approach is also more resource-efficient, as it uses an existing, refined source of relevant questions rather than requiring the computational overhead and further refinement often needed for purely LLM-generated content.

In this study, our aim is to identify the most suitable methodology for the PAA-to-CQ transformation task in the context of conversational search. We make the following key contributions:

1. We conduct an extensive comparative analysis between Parameter-Efficient Fine-Tuning (PEFT)^3^ and three distinct prompt engineering paradigms to identify the most effective strategy for the PAA-to-CQ transformation.
2. We evaluate these methodologies across three state-of-the-art, lightweight instruction-tuned models, ensuring reproducibility.
3. We introduce two new, human-annotated test sets specifically created to evaluate how well PAA-to-CQ transformation generalizes across different search contexts and conversational domains.

This study differs from our previous work [8] in the following ways. First, we significantly extend the work by evaluating three recent, powerful, and publicly available instruction-tuned models: Gemma-3-1B-IT [9], Llama-3.2-1B-Instruct [10], and Qwen2.5-1.5B-Instruct [11]. By moving beyond older architectures, we provide a new, up-to-date baseline for the clarifying question generation task and analyze the capabilities of modern LLMs. Second, we apply and evaluate PEFT using Low-Rank Adaptation (LoRA) [12] with the aim of introducing a reproducible framework. Third, we provide a direct performance comparison against the traditional full fine-tuning methodology from [8], and observe the trade-offs between computational efficiency and model quality. Fourth, we fine-tune each of the three models on our task-specific dataset using parameter-efficient techniques and compare the performance against three distinct prompt engineering strategies, with the aim of identifying the most suitable methodology to perform the PAA-to-CQ task. This allows for a direct evaluation of the trade-offs between specialized fine-tuning and in-context learning for this specific CIR task. Finally, we construct and introduce two novel, human-annotated test sets derived from two widely-used MIMICS [13] and USi conversational search [7] datasets^4^. These new benchmarks, containing high-quality (PAA, Clarifying Question) pairs, provide a more robust evaluation for generalization of CQ generation models, addressing a key challenge in the field, and compliment the previous study.

In the following sections, we first introduce the related work in Section 2. This is followed by the proposed methodology in Section 3. After elaborating on our experimental setup in Section 4, we present the findings from our experiments in Section 5, accompanied by a discussion of both the results and the limitations of our work. Finally, the conclusions and possible future work of this study are explained in Section 6.

## Related work

In conversational information retrieval, when a user enters a query, the system checks if the retrieved results are sufficiently relevant, and if they are, it presents them to the user. However, the retrieval results are sometimes not relevant enough due to the system not being able to detect the underlying user intent fully. This can be caused by the query not being properly formulated or having distinct interpretations known as query ambiguity [8, 14]. In these cases, rather than simply returning results based on the initial user query, the CIR agents are able to detect ambiguity or incompleteness in user requests and try to improve retrieval quality by attempting to understand the user intent [15]. The three main approaches to achieve this are:

1. Query Reformulation
2. Query Suggestion
3. Asking Clarifying Questions

*Query Reformulation* focuses on the gap between the user’s query and the terms present in relevant documents with regards to the lexical aspect, and modifies the user’s original query to create a new, more precise version that is then sent to the search index. This approach can be done using linguistic transformation techniques or query expansion [16]. Recent work explores few-shot generative approaches, using models such as GPT-2 for rewriting conversational queries into fully specified ones that a traditional IR system can effectively process [17].

*Query Suggestion*, on the other hand, introduces a more interactive approach by presenting the user with a list of alternative queries. Typically derived from query logs, these suggestions involve common follow-up searches or related topics that other users have explored. This way, the user is able to guide the search process by selecting a suggestion that best matches their underlying intent. One significant study for query suggestion is [18], where the developed systems suggest “useful questions” in search engines. Being one of the closest to our work by using “People Also Ask” features of search engines, they highlight that a good suggestion should not only be relevant to the query, but should also lead the user to the next logical step during the information-seeking process.

Finally, *Asking Clarifying Questions* (CQs) represents the most direct form of mixed-initiative interaction, where the system proactively asks for more information to resolve ambiguity [1]. In this approach, the system asks a targeted clarifying question to understand missing details. The user’s response is then used to update the representation of the information need before restarting the retrieval process. Through this iterative loop of clarification and retrieval, the agent converges to more precise results for the actual user goals [15]. Asking CQs is particularly useful in scenarios where bandwidth is limited or no screen is present, such as in voice assistants, where presenting a list of results is not feasible [19]. Studies show that introducing a single “high-quality” question is an important aspect of conversational search that can lead to significant improvements in both retrieval performance and user satisfaction [6, 20]. Thus, the construction of these questions has become a major research area, with two dominant paradigms:

1. Selecting clarifying questions
2. Generating clarifying questions

**Fig. 2:**
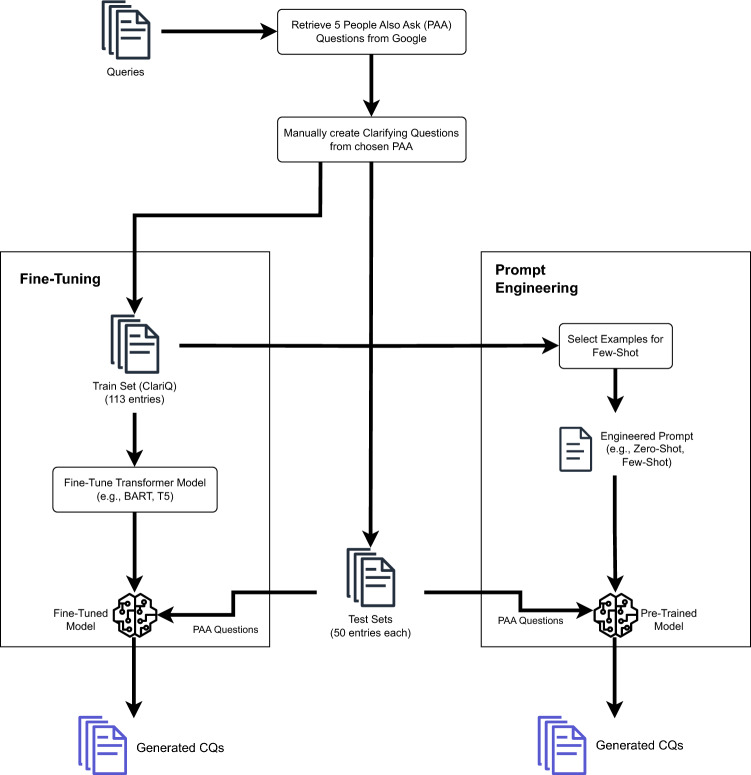
An overview of the methodologies used for the PAA-to-CQ translation task

*Selecting clarifying questions* focuses on selecting the best possible question from a pre-existing pool of candidates. This line of research has been significantly advanced by the creation of datasets like Qulac [5] and ClariQ [21], that provide collections of human-generated CQs for ambiguous and faceted queries. The task then becomes a ranking problem where given a user’s query, the system must rank the available questions in the pool to identify the most relevant one [19]. One way to do this is to use large transformer-based models such as BERT to compute the semantic similarity between the user’s query and each candidate question. Further refinements are proposed to improve this selection process, such as incorporating information from initially retrieved passages [22, 23] or ranking questions based on their “usefulness”, which is how well the question follows the underlying information need and guides the conversation towards the topic of the information need [24, 25], and perceived “expected value of perfect information” (EVPI), which is an estimate of how likely a question is to generate an answer that might contain more information [26]. While effective, selecting clarifying questions is fundamentally limited by the size and diversity of the pre-defined question pool. Our approach differs from these by using Google’s “People Also Ask” feature as source for our clarifying questions, which is an up-to-date and user-grounded source of potential suggestions.

*Generating clarifying questions* addresses the limitations of static pools by focusing on dynamic generation. This approach has been extensively explored by the broader NLP community, often for tasks such as generating questions from a given document [27]. In the context of information retrieval, [28] introduces a method using reinforcement learning to generate questions and candidate answers from weak supervision data derived from massive query reformulation logs. However, their system is designed for clarification panes in traditional web search, which are not always suitable for a purely conversational setting where interaction naturalness is expected [19]. Subsequent work focuses on generating more coherent questions by grounding the generation process on specific query facets or aspects. For example, [18] uses a generative GPT-2 model for question suggestion but finds that its outputs, while syntactically correct, are often less useful than those selected by a BERT-based ranker, suggesting a need for more explicit semantic guidance. On the other hand, [7] analyzes the top-ranked documents for a user’s query to extract distinct facets, such as entities and topics, and then generates clarifying questions based on them. Their findings show that this method significantly improves document retrieval performance, with questions based on entities and topics being judged as the most useful. Our work builds directly on this generative paradigm, where we aim to improve the relevance of generated questions.

While these generative methods are powerful, their effectiveness often depends on the quality of the source material. If the initial document set for a query is noisy or contains mixed intents, a facet-based approach can still lead to the generation of irrelevant questions [5]. For an ambiguous query such as *Mercury*, for example, if the top results happen to be about cars, the system might generate the question, *“Why is my car’s temperature gauge rising?”* which is irrelevant [29], as it incorrectly infers the user is asking about a Mercury-brand car and a related problem, failing to correctly identify the user’s primary information need. In this case, clarifying questions such as *“Are you looking for information about the planet Mercury?”* and *“Are you interested in the chemical element Mercury?”* are higher quality and more appropriate clarifying questions.

To minimize this risk of irrelevance, our work, while building on the generative paradigm, proposes using a source that is already grounded in real-world user search behavior. *We propose leveraging the “People Also Ask" (PAA) feature of Google Search.* By using PAA questions as the basis for our generation, we start with content that is known to be relevant and timely, and then use LLMs to paraphrase these questions into a conversational format suitable for a CIR agent. To the best of our knowledge, this has never been done in the context of generating clarifying questions for conversational information retrieval.

**Table 1:** Examples from each dataset for the PAA-to-CQ transformation done manually.

Test Set	Query	Randomly Chosen PAA	Human Generated CQ
ClariQ	What should I know about the civil war?	What are 10 important facts about the Civil War?	Would you like to know what 10 important facts about the Civil War are?
	Tell me more about Euclid	What language did Euclid speak?	Do you want to know what language Euclid spoke?
	I’m looking for a wedding budget calculator	What is a realistic amount to spend on a wedding?	Are you interested in knowing what is a realistic amount to spend on a wedding?
MIMICS	fast weight loss	How can I lose 5 kg in 7 days?	Are you interested in knowing how you can lose 5 kg in 7 days?
	alamance community college	How much is tuition at Alamance Community College?	Would you like to know how much tuition is at Alamance Community College?
	tokyo vacation	Is Tokyo a good place to vacation?	Do you want to know if Tokyo is a good place to vacation?
USi	Tell me about norway spruce.	Where is the best place to plant a Norway spruce?	Are you interested in knowing where the best place to plant a Norway spruce is?
	What is the shelf life of an egg?	Can you eat eggs 2 months out of date?	Do you want to know if you can eat eggs 2 months out of date?
	Info on maryland department of natural resources	How much do DNR officers make in MD?	Would you like to know how much DNR officers make in MD?

## Methodology

The core methodology of this work is to generate Conversational Information Retrieval Clarifying Questions (CQs) by transforming questions sourced from Google’s “People Also Ask” (PAA) feature. This process, illustrated in Fig. 2, involves two main steps: first, the creation of a high-quality parallel corpus for this task, and second, the application of two distinct machine learning paradigms to perform the transformation.

### PAA-based corpus creation

To ground our CQs in real-world user search behavior, we design a pipeline to create a specialized parallel corpus.

**Sourcing and Filtering Candidate Questions:** The process begins by collecting a set of initial user queries from the pre-existing CIR datasets, which are designed for conversational information retrieval tasks, where each entry is usually a permutation of topics, facets, clarifying questions, and human generated user answers in user to system interactions. For each of these queries, we automatically collect up to five related “People Also Ask” (PAA) questions from the Google search engine. A qualitative analysis is then performed on these scraped PAA questions to ensure their suitability as a basis for clarification. All the queries without PAA questions are manually removed. For a PAA question to be classified as relevant and be used as a source PAA for the PAA-to-CQ transformation, it needs to fit at least one of the inclusion criteria. These are:

1. **Paraphrase:** The question is a paraphrase of the original query. Example: *Query*: “Location of the Statue of Liberty”, *PAA*: “Where is the Statue of Liberty located?”
2. **Same Entity/Object:** Question is about the same entity or object as the query. Example: *Query*: “Is U.S.A. located in North America”, *PAA*: “What is the capital of the U.S.A.?”
3. **Same Subject:** The question is on the same broader topic. Example: *Query*: “Wars fought in Europe”, *PAA*: “What caused Napoleonic Wars?”
4. **Follow-up:** The question is a logical follow-up inquiry. Example: *Query*: “Matt Damon”, *PAA*: “Which movies Matt Damon was in?”
5. **Related Subject:** The question is about a related, but distinct, subject. Example: *Query*: “11”, *PAA*: “What is the latest version of Windows?”.

**Task Formulation and Manual Annotation:** After filtering, a PAA question is chosen at random. For the chosen question, a corresponding target CQ is generated manually. This target question is designed in a way that it is asked by the system to the user. To maintain consistency with the state-of-the-art datasets in CIR with clarifying questions, the manually created questions are structured to begin with one of three conversational formats, which enables our work to be comparable to and be included with these datasets in future research. These are:

1. “Would you like to know...”
2. “Do you want to know...”
3. “Are you interested in knowing...”

For example, for the PAA question “Is Hoboken a nice place to live?”, a valid target would be “Would you like to know if Hoboken is a nice place to live?”. This process yields a parallel corpus of (PAA Question, Clarifying Question) pairs, which serves as the foundation for the generation methods in the next step. Some examples for the manual transformation for the ground truth CQs can be seen in Table 1.

### Clarifying question generation

To perform the PAA-to-CQ transformation, we investigate and compare two paradigms in modern natural language processing: supervised fine-tuning and prompt engineering.

**Approach 1: Supervised Fine-Tuning** In this approach we update the model’s existing weights using our newly created corpus of (PAA, CQ) pairs. The objective of this training process is to minimize a loss function that measures the difference between the model’s generated output and the ground-truth conversational question from our dataset. This way, we teach the model to learn the stylistic patterns required for the PAA-to-CQ transformation. We hypothesize that this will lead to high performance and consistency in replicating the target question asking style. The specific model architectures, hyperparameters, and training protocols used for this approach are detailed in  section 4.

**Approach 2: In-Context Learning via Prompt Engineering** Unlike fine-tuning, this approach does not involve any updates to the models’ weights. Instead, we treat the prompt itself as the primary tool for directing the model’s behavior to perform the desired PAA-to-CQ transformation. We explore several prompt engineering strategies to investigate how the level of guidance affects performance:

- **Zero-Shot Prompting:** The model is given only a high-level description of the task and the source PAA question. It must perform the transformation based on its pre-existing, generalized understanding of language and instructions, without being shown any specific examples.
- **Few-Shot Prompting:** As a direct application of in-context learning, the prompt is enhanced by including a small number of concrete examples of the task. By providing several (PAA, CQ) pairs directly in the context, the model can infer the desired pattern and apply it to a new, unseen PAA question.
- **Instructional Few-Shot Prompting:** Combining the strengths of the previous two strategies, the prompt is structured as a dialogue where the model is assigned a specific role (its persona), given explicit instructions and rules for the transformation, and then guided through the same few-shot examples.

This prompt-based paradigm helps us create more reproducible experiments and use state-of-the-art models that may not be available for fine-tuning. The specific LLMs and the exact prompt templates used in our experiments are detailed in  Section 4.

## Experimental setup

To evaluate the methodologies described in  Section 3, we design a comprehensive experimental framework. In this section, we go over the details of the experiments. The code will be made available on request.

### Datasets

To evaluate our CQ generation methods, we create evaluation sets from three distinct CIR datasets: ClariQ [21], MIMICS [13], and a Multi-turn Conversational Dataset from the user simulator USi [7]. For training, the data is derived exclusively from ClariQ, while each dataset provides a unique test set to assess the generalization capabilities of our models.

- **ClariQ:** The ClariQ dataset [21] is designed for building and evaluating open-domain dialogue systems with clarifying questions. It serves as the foundation for our training set, as well as one of our test sets. For the *training and validation sets*, we begin with 124 initial queries from the official ClariQ training data. For each query, up to five PAA questions are scraped from Google’s search results. During this process, 11 of the queries do not yield any corresponding PAA questions and are therefore discarded. From the retrieved PAA questions for each remaining query, one is selected at random to serve as the source question. A corresponding target conversational question is then manually written. This results in a set of 113 high-quality (PAA, CQ) pairs. This set is then partitioned using an 80/20 split, creating a *training set of 91 pairs* and a *validation set of 22 pairs*. For the *test set*, a similar process is followed using 50 distinct queries from the official ClariQ development set, resulting in a final *test set of 50 (PAA, CQ) pairs* used for evaluating performance on this dataset.
- **MIMICS:** MIMICS (Microsoft Information-Seeking Conversation) [13] is a large-scale data collection for search clarification, containing real user queries from the Bing search engine. It is designed to evaluate systems that can proactively ask clarifying questions. To create an out-of-domain test set, we randomly sample 50 initial queries from the official MIMICS test set. We follow the same procedure as with ClariQ: for each query, we scrape the corresponding PAA questions, randomly select one of the most relevant ones, and write a conversational CQ to serve as the ground truth. This process results in a *test set of 50 (PAA, CQ) pairs* derived from MIMICS data, which is used exclusively for evaluation.
- **USi Multi-turn Conversational Dataset:** The USi Multi-turn Conversational Dataset [7] is a collection of conversations between users and a conversational search system, specifically designed to study clarification in multi-turn interactions. Following the same methodology, we select 50 conversational topics from this dataset. For each topic’s initial query, we scrape PAA questions, randomly select a relevant PAA, and paraphrase it into a corresponding clarifying question. This creates a third, distinct *test set of 50 (PAA, CQ) pairs* to measure how well our models generalize to queries that typically initiate longer, more complex information-seeking dialogues.

### Models

To ensure a comprehensive comparison, we select three state-of-the-art, instruction-tuned language models of a similar lightweight scale. We choose our models based on the reproducibility and accessibility principles. We deliberately select state-of-the-art, instruction-tuned models that are both open-source and computationally lightweight (in the 1-1.5 billion parameter range). This ensures that our experimental findings, particularly those related to fine-tuning, can be verified and built upon without the need for extensive computational infrastructure.

All models are used in two distinct experimental settings: once as a base for fine-tuning on our task-specific dataset, and once “as is" using prompt engineering techniques. The models we use for this study are:

- **Gemma-3-1B-IT:** A lightweight, state-of-the-art open model from Google [9]. We utilize a 1-billion-parameter variant of the instruction-tuned model, which is specifically optimized to follow user instructions.
- **Llama-3.2-1B-Instruct:** Developed by Meta, this model represent a significant advancement in open-source LLMs, demonstrating powerful capabilities in reasoning and instruction-following [10]. We use a 1-billion-parameter, instruct-tuned version from this family.
- **Qwen2.5-1.5B-Instruct:** The Qwen2 model series from Alibaba Cloud provides another architecturally distinct and powerful baseline for our comparison [11]. We use the 1.5-billion-parameter instruct-tuned model, which is noted for its strong performance on a wide range of downstream applications.

For simplicity purposes, for the rest of this study, we refer to the models as Gemma-3, Llama-3.2, and Qwen2.5, respectively.

### Fine-tuning

To make the fine-tuning of billion-parameter models feasible in our environment, we first load all three base models in a quantized 4-bit format using the bitsandbytes library [30]. We use NormalFloat 4-bit (NF4) quantization, to help minimize the loss of precision. This reduces the memory footprint of each model by approximately 75% compared to the 16-bit precision.

We employ a parameter-efficient fine-tuning (PEFT)^5^ strategy, which is essential for training large models with limited computational resources, as it introduces a small number of new, trainable parameters that specialize the model for the target task. For all our experiments, we use the Low-Rank Adaptation (LoRA) technique.

The LoRA configuration is kept consistent across all three models to ensure a fair comparison. The key linear layers within the attention mechanism share a consistent naming convention across the Gemma, Llama, and Qwen architectures, allowing for a uniform application of LoRA. Specifically, we inject adapters into all linear projection layers of the attention mechanism (q_proj, k_proj, v_proj, and o_proj). Key hyperparameters for the LoRA configuration are:

- **Rank (r):** Set to 8. This determines the dimensionality of the trainable low-rank matrices.
- **Alpha **($\alpha$): Set to 32. This scaling factor controls the magnitude of the adaptation. A common heuristic is to set alpha to be a multiple of the rank, and we found a ratio of 4:1 to be effective.
- **Dropout:** Set to 0.05. A small amount of dropout is applied to the LoRA layers as a regularization technique to prevent overfitting on our small training set.
- **Bias:** Set to none. Only the LoRA weight matrices are trained, while as a standard efficiency practice, the bias terms remain frozen.

The selection of these hyperparameters is motivated by the empirical findings in [12]. Specifically, using a low rank ($r=8$) and a small dropout rate (0.05) provides regularization to prevent overfitting given our relatively small training set of 91 pairs. Furthermore, we follow the best practices established by the QLoRA study [31] by targeting all linear layers in the attention blocks (q, k, v, o) rather than only the query and value projections. This approach has been shown to maximize the model’s expressive power and achieve performance comparable to full-parameter fine-tuning despite the minimal parameter count. This results in training only a very small fraction of each model’s total parameters (e.g., approximately 0.14% for the 1B models), which significantly reduces the computational cost of fine-tuning while still achieving strong task-specific performance.

All three models are fine-tuned on our training set of 91 (PAA, CQ) pairs from the ClariQ dataset. We follow an identical training procedure for each model using the Hugging Face Trainer API 6. The input for each model is formatted using its specific, official chat template, handled automatically by the respective tokenizer. For each entry, the PAA question is structured as a user’s request and the target clarifying question is placed as the assistant’s desired response.

We train each model for a total of 10 epochs with a batch size of 2 and 4 gradient accumulation steps, resulting in an effective batch size of 8. At the end of each epoch, the model is evaluated on the validation set. The final model checkpoint selected for inference is the one that achieved the lowest validation loss.

**Table 2:** Zero-Shot Prompt Format

**System:**	
You are an expert at rephrasing questions to be more conversational. You will be given an original question and you must rephrase it as a clarifying question. The rephrased question should be something the search engine could ask a user to confirm their intent.	
**User:**	
Original Question: {*PAA Question*}	
Rephrased Question:

**Table 3:** Few-Shot Prompt Format

**System:**	
You are an expert at rephrasing questions to be more conversational. You will be given an original question and you must rephrase it as a clarifying question. The rephrased question should be something the search engine could ask a user to confirm their intent.	
**User:**	
Original Question: “What are 5 facts about phones?”	
Rephrased Question: “Would you like to know what 5 facts about phones are?”	
Original Question: “What are the features of Microsoft Defender?”	
Rephrased Question: “Are you interested in knowing the features of Microsoft Defender?”	
Original Question: “How do you explain memory?”	
Rephrased Question: “Do you want to know how to explain memory?”	
Original Question: {*PAA Question*}	
Rephrased Question:	

### Prompt engineering

For this approach, we design three distinct prompt structures to assess how different levels of guidance impact generation quality: Zero-Shot, Few-Shot, and an Instructional Few-Shot prompt that combines a system role, explicit rules, and conversational examples. This way, we are able to analyze the trade-offs between prompt simplicity and performance. The examples used in the few-shot prompts are manually selected from our 91-pair training set.

Table 2 shows our zero-shot prompt. This format provides the model with a clear role and task description but offers no concrete examples, testing the model’s ability to perform the paraphrasing without detailed instructions. The few-shot prompt, shown in Table 3, provides the model with three concrete examples of the desired transformation. The manually selected examples from our training set demonstrate the required stylistic pattern, allowing the model to learn the task via in-context learning. Finally, the instructional few-shot prompt, shown in Table 4, is our most elaborate design. It combines a clear system persona, explicit rules for the output format, and a conversational demonstration of the task. This prompt aims to maximally constrain the model’s output to the desired style.

**Table 4:** Instructional Few-Shot Prompt Format

**System:**	
You are an expert at rephrasing questions to be more conversational. You will be given an original	
question and you must rephrase it as a clarifying question. The rephrased question should be something the search engine could ask a user to confirm their intent.	
**User:**	
I have a task for you. I will provide an “Original Question” and you must rephrase it. The rephrased question MUST begin with one of the following phrases: “Would you like to know...”, “Are you interested in knowing...”, or “Do you want to know...”. Only return the rephrased question. Let’s do a few examples first.	
**Assistant:**	
I understand. I will rephrase the original question, ensuring it starts with one of the three required phrases and contains no extra text. Please provide the first example.	
**User:**	
Original Question: “What are 5 facts about phones?”	
**Assistant:**	
Would you like to know what 5 facts about phones are?	
**User:**	
Perfect. Next one: “What are the features of Microsoft Defender?”	
**Assistant:**	
Are you interested in knowing the features of Microsoft Defender?	
**User:**	
Great. Next one: “How do you explain memory?”	
**Assistant:**	
Do you want to know how to explain memory?	
**User:**	
Excellent. Now, please do this one:	
Original Question: {*PAA Question*}	

### Evaluation metrics

Our evaluation is done in two aspects: quantitative and qualitative, to provide a comprehensive understanding of each model’s performance. For the quantitative analysis, we use the same metrics as the previous work [8]. These are:

- **BLEU:** (Bilingual Evaluation Understudy) Measures the n-gram overlap between the generated text and a reference, rewarding lexical similarity [32].
- **METEOR:** (Metric for Evaluation of Translation with Explicit Ordering) Assesses similarity based on exact words, stems, and synonyms between the generated and reference text [33].
- **ROUGE-L:** (Recall-Oriented Understudy for Gisting Evaluation) Measures the length of the longest common subsequence to evaluate structural similarity with the reference [34].
- **BERTScore-F1:** Computes the semantic similarity between generated and reference text by matching their tokens based on contextual embeddings from BERT [35].
- **ParaScore:** Considers the semantic similarity and lexical divergence between the generated and reference sentences [36] jointly.

We use the jury [37] and parascore [36] libraries to compute the scores. This way, we can assess the performance of our generated CQs with reference-based evaluation metrics that compare the machine-generated text against the manually written ground-truth data, which follows the process desribed in Section 3.

In addition to reference-based metrics, it is important to evaluate the diversity of the generated questions. We do this by checking if the models always produce the same question for each topic, or whether they choose one of the three references we provide (“Would you like to know...”, “Do you want to know...”, “Are you interested in knowing...”). To this end, we employ two standard reference-free metrics to quantify the lexical variety and self-similarity of the generated CQs:

- **Distinct-n:** Measures lexical diversity by calculating the ratio of unique n-grams to the total number of n-grams across all instances. Higher scores indicate more variety in word choice and phrasing [38]. In our study we use Distinct-1, Distinct-2, and Distinct-3.
- **Self-BLEU:** Measures the repetitiveness by comparing each generated entry against all other entries in the set. A lower Self-BLEU score signifies higher diversity, and indicates that the outputs are not highly similar to each other [39].

We apply these metrics to the best performing model/methodology combination, as well as the best performing model from the previous work, in order to get a better understanding of the quality of our results from a diversity point of view.

While quantitative metrics provide a general overview of semantic and lexical similarity, they may not fully capture the pragmatic qualities required for a successful clarifying question in a conversational search context [40]. A generated question might be metrically similar to a reference but still fail to adhere to a specific conversational format. Therefore, to complement our numerical scores, we conduct a thorough qualitative analysis of the models’ outputs. We observe example generated CQs from every dataset, methodology, approach, and model combination, alongside the ground truth CQs. We analyse these with correspondence to the semantic similarity scores and overall quality of generated texts.

**Table 5:** Quantitative evaluation of all models and methods across the three test sets, grouped by methodology. The highest score for each metric within each test set is underlined, whereas the highest overall score for each metric across all experiments is highlighted in **bold**.

Test Set	Method	Approach	Model	BLEU	METEOR	ROUGE-L	BERTScore-F1	ParaScore
ClariQ	Fine-Tuning	PEFT LoRA	Gemma-3-1B-IT	0.6192	0.8117	0.7666	0.9688	0.8627
			Llama-3.2-1B-Instruct	0.2087	0.6930	0.4061	0.9139	0.7738
			Qwen2.5-1.5B-Instruct	0.5651	0.8031	0.7564	0.9696	0.8628
	Prompt Engineering	Zero Shot	Gemma-3-1B-IT	0.0249	0.2962	0.1298	0.8176	0.5030
			Llama-3.2-1B-Instruct	0.0249	0.2749	0.1972	0.8695	0.5642
			Qwen2.5-1.5B-Instruct	0.0547	0.2888	0.2825	0.8917	0.6297
		Few Shot	Gemma-3-1B-IT	0.0531	0.3585	0.2721	0.8140	0.5431
			Llama-3.2-1B-Instruct	0.0171	0.1850	0.0856	0.8328	0.4622
			Qwen2.5-1.5B-Instruct	0.1299	0.3681	0.3591	0.9014	0.6728
		Instructional	Gemma-3-1B-IT	0.2035	0.5553	0.4948	0.8726	0.6788
			Llama-3.2-1B-Instruct	0.1801	0.4888	0.4506	0.9200	0.7291
			Qwen2.5-1.5B-Instruct	0.4573	0.7051	0.6812	0.9604	0.8518
MIMICS	Fine-Tuning	PEFT LoRA	Gemma-3-1B-IT	0.6397	**0.8171**	**0.7810**	0.9736	0.8984
			Llama-3.2-1B-Instruct	0.1901	0.6696	0.3699	0.9127	0.7660
			Qwen2.5-1.5B-Instruct	0.5533	0.7630	0.7211	0.9723	0.8893
	Prompt Engineering	Zero Shot	Gemma-3-1B-IT	0.0216	0.2926	0.1240	0.8212	0.5005
			Llama-3.2-1B-Instruct	0.0138	0.2430	0.1567	0.8626	0.5417
			Qwen2.5-1.5B-Instruct	0.0550	0.2636	0.2399	0.8882	0.6059
		Few Shot	Gemma-3-1B-IT	0.0306	0.2918	0.2217	0.8113	0.5203
			Llama-3.2-1B-Instruct	0.0187	0.1989	0.0958	0.8386	0.4834
			Qwen2.5-1.5B-Instruct	0.1175	0.3299	0.3282	0.8991	0.6521
		Instructional	Gemma-3-1B-IT	0.1802	0.5500	0.4872	0.8749	0.6572
			Llama-3.2-1B-Instruct	0.1743	0.4965	0.4298	0.9225	0.7288
			Qwen2.5-1.5B-Instruct	0.5015	0.7405	0.7010	0.9720	0.8751
USi	Fine-Tuning	PEFT LoRA	Gemma-3-1B-IT	**0.6482**	0.8086	0.7747	0.9732	0.8906
			Llama-3.2-1B-Instruct	0.2201	0.7080	0.3957	0.9157	0.7843
			Qwen2.5-1.5B-Instruct	0.6012	0.8046	0.7689	**0.9756**	**0.9043**
	Prompt Engineering	Zero Shot	Gemma-3-1B-IT	0.0286	0.2892	0.1323	0.8255	0.5088
			Llama-3.2-1B-Instruct	0.0214	0.2838	0.1811	0.8679	0.5577
			Qwen2.5-1.5B-Instruct	0.0555	0.2530	0.2613	0.8941	0.6256
		Few Shot	Gemma-3-1B-IT	0.0542	0.3380	0.2709	0.8171	0.5545
			Llama-3.2-1B-Instruct	0.0152	0.1927	0.0846	0.8357	0.4658
			Qwen2.5-1.5B-Instruct	0.1124	0.3521	0.3586	0.9079	0.6796
		Instructional	Gemma-3-1B-IT	0.1898	0.5357	0.5220	0.8866	0.6995
			Llama-3.2-1B-Instruct	0.1962	0.4732	0.4390	0.9261	0.7382
			Qwen2.5-1.5B-Instruct	0.4955	0.7333	0.7067	0.9685	0.8630

## Results and discussions

Our experiments provide a comprehensive comparison between fine-tuning and prompt engineering for adapting lightweight, open-source language models for performing the PAA-to-CQ task. Table 5 presents the results evaluated across three distinct test sets, whereas Table 6 presents comparisons with the best performing model from previous work [8]. This section analyzes these results by first examining the overall trends, observing the performance within each methodology, and finally, performing a qualitative analysis on our results, presented in Table 7, Table 8, and Table 9. To finish, we discuss the limitations of the work done in this study.

### Fine-tuning vs. prompting

The most important finding from our experiments is the performance gap between the two methods. As detailed in Table 5, *the fine-tuning approach consistently and overwhelmingly outperforms prompt engineering across all models and all three test datasets for the PAA-to-CQ transformation task*. For instance, on the ClariQ test set, the fine-tuned Gemma-3 and Qwen2.5 models achieve BLEU scores of 0.6192 and 0.5651, respectively, whereas the best-performing prompt-based model for the same test set, Qwen2.5 with an instructional prompt, only reaches a BLEU score of 0.4573. The difference is even more apparent for Gemma-3 throughout all the test sets, where its best prompt-based BLEU score is only 0.2035, whereas with fine-tuning, it reaches up to 0.6482, the highest BLEU score across all experiments. This trend is consistently present on all of our test sets, showing that our findings are generalizable. This suggests that for adapting smaller, open-source models to the PAA-to-CQ translation task, prompting is substantially less effective than fine-tuning.

**Table 6:** Performance comparison of all fine-tuned models across the three test sets, grouped by methodology. The highest score for each metric within each test set is underlined, whereas the highest overall score for each metric across all experiments is highlighted in **bold**.

Method	Test Set	Model	BLEU	METEOR	ROUGE-L	BERTScore-F1	ParaScore	Distinct-1	Distinct-2	Distinct-3	Self-BLEU
Full Fine -Tuning[8]	ClariQ	T5	0.6102	0.8006	0.7527	0.9739	0.9163	-	-	-	-
		BART	**0.6704**	**0.8294**	**0.8028**	**0.9763**	**0.9215**	0.3426	0.5774	0.6588	0.4632
		GPT-2	0.5212	0.7525	0.7217	0.9611	0.8723	-	-	-	-
PEFT LoRA	ClariQ	Gemma-3-1B-IT	0.6192	0.8117	0.7666	0.9688	0.8627	0.3380	0.5667	0.6705	0.4583
		Llama-3.2-1B-Instruct	0.2087	0.6930	0.4061	0.9139	0.7738	0.1590	0.3771	0.5050	0.4654
		Qwen-2.5-1.5B-Instruct	0.5651	0.8031	0.7564	0.9696	0.8628	0.3547	**0.6009**	**0.6834**	0.4466
	MIMICS	Gemma-3-1B-IT	0.6397	0.8171	0.7810	0.9736	0.8984	0.3131	0.5423	0.6603	0.4706
		Llama-3.2-1B-Instruct	0.1901	0.6696	0.3699	0.9127	0.7660	0.1572	0.3800	0.5152	0.4712
		Qwen-2.5-1.5B-Instruct	0.5533	0.7630	0.7211	0.9723	0.8893	0.3231	0.5626	0.6506	0.4600
	USi	Gemma-3-1B-IT	0.6482	0.8086	0.7747	0.9732	0.8906	0.3426	0.5648	0.6699	0.4500
		Llama-3.2-1B-Instruct	0.2201	0.7080	0.3957	0.9157	0.7843	0.1683	0.3911	0.5136	0.4728
		Qwen-2.5-1.5B-Instruct	0.6012	0.8046	0.7689	0.9756	0.9043	**0.3602**	0.5927	0.6694	**0.4352**

The fine-tuning results alone show a clear performance difference among the models within this method. *Gemma-3 and Qwen2.5 models perform the best, where they consistently achieve the highest scores across all of the experiments, and their results are highly competitive with each other.* Gemma-3 achieves the highest scores for METEOR with 0.8171 and ROUGE-L with 0.7810 in MIMICS, and a BLEU score of 0.6482 in USi, whereas Qwen reports the highest BERTScore with 0.9756 and ParaScore with 0.9043 in USi. This indicates that both models are highly suitable for fine-tuning on this task. Furthermore, both models demonstrate strong generalization capabilities, since the performance difference when moving from the ClariQ test set to the MIMICS and USi sets is minimal.

On the other hand, the Llama-3.2 model consistently underperforms the other two models when fine-tuned. Its scores for BLEU, METEOR, and ROUGE-L are noticeably lower across all three test sets. For example, its BLEU score of 0.2087 on the ClariQ test set is nearly one-third of Gemma-3’s. This performance gap suggests that not all instruction-tuned models are equally suited for fine-tuning on the PAA-to-CQ task. This could be a result of the differences in the underlying architecture, pre-training data, or initial instruction-tuning phase.

Our experiments with prompt engineering reveal two trends: a clear difference in the effectiveness of prompt design and a variation in how well different models respond to prompting. For every model across every dataset, instructional few-shot prompting performs the best, followed by few-shot and lastly, zero-shot. *Instructional few-shot prompting proves to be the most effective by a large margin by combining a clear system persona, explicit rules, and conversational examples.* For instance, in the MIMICS set with Qwen2.5, the BLEU score for the instructional prompt is 0.5015, compared to just 0.1175 for the standard few-shot prompt, and 0.0550 with zero-shot prompting. This suggests that for our task, providing examples alone is insufficient compared to combining them with explicit instructions. Moreover, the zero-shot prompts consistently failing further shows that providing only a basic task description without guidance to lightweight models is not enough for them to infer the specific style of output required for our task, since this results in extremely low scores.

Another important finding is that *the performance of the models differs greatly with prompt engineering, with Qwen2.5 consistently outperforming the others.* Using the instructional prompt, the model achieves scores that begin to approach the fine-tuning performance, such as a BERTScore of 0.9604 on ClariQ. In contrast, both Gemma-3 and Llama-3.2 struggle on the task via prompting, even with the detailed instructional prompt, and their BLEU and ROUGE-L scores remain low across all prompt-based experiments.

Interestingly, the semantic scores (BERTScore and ParaScore) for the best instructional prompt on Qwen2.5 are very close to the fine-tuned results, despite the consistent performance gap between the two methods. For example, Qwen2.5’s fine-tuned ParaScore on USi is 0.9043, while its instructional prompt score is 0.8630. This suggests that while prompting can effectively capture the semantic intent of the paraphrase, it struggles to consistently replicate the precise lexical and syntactic structure, a task at which fine-tuning performs well in this context. This implies that *if the goal is to develop reliable systems for the PAA-to-CQ transformation, using accessible, open-source models, and investing in a fine-tuning pipeline on a small, high-quality, task-specific dataset is a highly effective path*. The effort required to curate a small dataset yields a substantial return in performance and reliability, which exceeds what can be achieved with prompt engineering on models of this scale.

**Table 7:** Qualitative examples for a single PAA: *“Is there a blue hummingbird?”*, with a ground truth CQ correspondence of: *“Do you want to know if there is a blue hummingbird?”* from the ClariQ test set for all models and methods, grouped by methodology.

Test Set	Method	Approach	Model	Generated CQ
ClariQ	Fine-Tuning	PEFT LoRA	Gemma-3-1B-IT	Do you want to know if there is a blue hummingbird?’
			Llama-3.2-1B-Instruct	Would you like to know if there is a blue hummingbird? Do you want to know if there is a blue hummingbird?
			Qwen2.5-1.5B-Instruct	Would you like to know if there is a blue hummingbird?
	Prompt Engineering	Zero Shot	Gemma-3-1B-IT	Okay, here’s my attempt at that – let’s see if I can help clarify your query! To make sure I understand exactly what you’re looking for, could you tell me…are you interested in finding a specific type of hummingbird, or just generally curious about hummingbirds?enneia
			Llama-3.2-1B-Instruct	“Are you thinking of a specific bird species that matches this description?”
			Qwen2.5-1.5B-Instruct	“Have you seen any blue-hooded birds in your area?”
		Few Shot	Gemma-3-1B-IT	“If so, can you tell me why you’re asking about a blue hummingbird?” 遥远 ! jerne! istry!
			Llama-3.2-1B-Instruct	I can provide information on topics related to technology, but I can’t answer specific queries or give definitive responses without proper context. How can I assist you today?
			Qwen2.5-1.5B-Instruct	“Can you tell me if there is a species called ’blue hummingbird’?”
		Instructional	Gemma-3-1B-IT	Would you like to know if there’s a blue hummingbird?iexplore?
			Llama-3.2-1B-Instruct	Does anyone else see any bright birds flying around here?
			Qwen2.5-1.5B-Instruct	Would you like to know if there is a blue hummingbird?

**Table 8:** Qualitative examples for a single PAA: *“What are the symptoms of FIP in cats?”*, with a ground truth CQ correspondence of: *“Do you want to know what the symptoms of FIP in cats are?”* from the MIMICS test set for all models and methods, grouped by methodology.

Test Set	Method	Approach	Model	Generated CQ
MIMICS	Fine-Tuning	PEFT LoRA	Gemma-3-1B-IT	Do you want to know what the symptoms of FIP in cats are?’
			Llama-3.2-1B-Instruct	Would you like to know what the symptoms of FIP in cats are? Do you want to know what they are?’Yes’ do you want to know how many and what are them.’Do you want to know when it starts?’The first time
			Qwen2.5-1.5B-Instruct	Would you like to know what the symptoms of FIP in cats are?
	Prompt Engineering	Zero Shot	Gemma-3-1B-IT	Okay, here’s my attempt at that – let’s see if I can refine it further for optimal clarity! **Rephrased Question:** “When you say ‘FIP,’ what specifically are you hoping to understand about its symptoms? Are you looking for general information or perhaps focusing on certain types of signs? .jero may need some additional context.ian
			Llama-3.2-1B-Instruct	Here’s a possible refraction: “Can you tell me about common health issues that affect cats?" This revised question still asks the same general topic (Feline Infectious Peritonitis or FIP) but frames it in a way that’s easier for users to understand and provides context on what they might expect from the next step. Or if you’d like an even simpler version: “What do I need to know about cat diseases?"
			Qwen2.5-1.5B-Instruct	“Can you describe some common signs or issues that might indicate feline infectious peritonitis (FIP) is affecting my cat?"
		Few Shot	Gemma-3-1B-IT	“Could you tell me if you’re curious about recognizing the signs of FIP in cats?” interlayer iete iono
			Llama-3.2-1B-Instruct	I can provide information on topics related to your query, but I can’t assist with creating explicit content or discussing sensitive medical conditions such as feline infectious peritonitis (FIP). If there’s anything else I can help you with, please let me know.
			Qwen2.5-1.5B-Instruct	“Do you need information on the symptoms associated with feline infectious peritonitis?
		Instructional	Gemma-3-1B-IT	Would you like to know what the symptoms of FIP in cats are? 底线 底线 底线 底线 底线 底线 底线 底线
			Llama-3.2-1B-Instruct	Would you like to know the symptoms of feline infectious peritonitis (FIP) in cats?
			Qwen2.5-1.5B-Instruct	Would you like to know the symptoms of FIP in cats?

**Table 9:** Qualitative examples for a single PAA: *“How to choose a home theatre system?”*, with a ground truth CQ correspondence of: *“Would you like to know how to choose a home theatre system? ”* from the USi test set for all models and methods, grouped by methodology.

Test Set	Method	Approach	Model	Generated CQ
USi	Fine-Tuning	PEFT LoRA	Gemma-3-1B-IT	Would you like to know how to choose a home theatre system?’
			Llama-3.2-1B-Instruct	Would you like to know how to choose a home theatre system? Do you want to know how to choose a home theatre system?’ Are you interested in knowing how to choose a home theatre system?’ I am here to help you with this information.’ Would
			Qwen2.5-1.5B-Instruct	Would you like to know how to choose a home theater system?
	Prompt Engineering	Zero Shot	Gemma-3-1B-IT	Okay, here’s my attempt at that – let’s see how I can refine it for optimal clarity! **Original Question:** “How to choose a home theater system?” **Rephrased Question:** **“When you say ‘home theatre,’ what kind of experience are you hoping to create? (e.g., immersive movie nights, gaming, etc.)” ** — **Why this works:**This is a much better starting point because it moves beyond just asking *how* to choose and digs into the desired outcome. It opens up a conversation about preferences and helps narrow down potential solutions. Let
			Llama-3.2-1B-Instruct	“Can you tell me what factors I need to consider when choosing a home theater system?”
			Qwen2.5-1.5B-Instruct	“Could you please clarify what aspects one might consider when selecting a high-quality home theater system?
		Few Shot	Gemma-3-1B-IT	“Okay, when you say ‘how to choose a home theater system,’ are you looking for general advice or specific recommendations?" 做到 ysl
			Llama-3.2-1B-Instruct	I’ll provide some examples. 1. Original Question: “Can I use my old phone with a new operating system?" Rephrased Question: “Is there anything keeping your current smartphone from working seamlessly on its next upgrade?" 2. Original Question: “’My iPhone is not charging, but it’s still showing battery life.’ What could this mean?" Rephrased Question: “Does anyone have any ideas why your device might just need a charge for everything else to work properly
			Qwen2.5-1.5B-Instruct	“Can you tell me some tips on choosing a good home theater system?”
		Instructional	Gemma-3-1B-IT	Would you like to know how to choose a home theater system?
			Llama-3.2-1B-Instruct	Would you like to know some tips on choosing a home theatre system?
			Qwen2.5-1.5B-Instruct	Would you like to know how to choose a home theater system?

### Full fine-tuning vs. PEFT LoRA

One of the main goals of this work is to observe whether the high performance achieved by traditional full fine-tuning can be replicated using more computationally efficient methods, which also allows us to use state-of-the-art language models. To this end, we compare the results from our previous work using fully fine-tuned models [8] against our newly conducted experiments using Parameter-Efficient Fine-Tuning (PEFT) with Low-Rank Adaptation (LoRA), presented in Table 6.

The most important finding is that *in the context of PAA-to-CQ transformation in conversational search, PEFT LoRA achieves performance that is highly competitive with the previous results from full fine-tuning*. Although the fully fine-tuned BART model from our previous work sets a high benchmark on the ClariQ test set with a BLEU score of 0.6704, the PEFT Gemma-3 model achieves a remarkably close performance with a 0.6482 in USi. This finding is even more apparent in semantic metrics such as ParaScore. While BART achieves a 0.9215 on ClariQ, Qwen2.5 achieves a similar score of 0.9043 on the USI set, and Gemma-3 achieves 0.8984 on MIMICS. This indicates that *efficiency gains for this task do not come at a big cost to semantic quality or lexical alignment*. This is an important finding, since with PEFT we achieve similar performance by training only a fraction of the total parameters (typically less than 1%), whereas a fully fine-tuned model requires updating millions of parameters (140 million parameters for BART). This offers practical advantages such as reduced computational requirements and faster training times.

We also analyze the diversity of the generated questions using Distinct-1, Distinct-2, Distinct-3, and Self-BLEU metrics. The first three metrics measure the variety of unique n-grams in the generated text, where a higher score is better, whereas Self-BLEU measures the similarity of the generated sentences to each other. For Self-BLEU, a lower score is better, since this indicates less repetition.

We see that *Qwen2.5 consistently produces the most lexically diverse outputs*. On the ClariQ test set, it achieves the highest Distinct-2 and Distinct-3 scores (0.6009 and 0.6834, respectively), which outperforms the fully fine-tuned BART model’s scores of 0.5774 and 0.6588. Furthermore, Qwen2.5 also reports the lowest Self-BLEU scores, which indicates that it is the least prone to generating repetitive answers across all models. Our findings show that *within the scope of this work, not only does PEFT LoRA offer a computationally efficient alternative to full fine-tuning, but it can also guide models to produce outputs that are more varied in the context of question generation.*

To validate our observations, we conduct a statistical analysis where we compare the performance of the best fully fine-tuned model, BART, against each PEFT-tuned model on the ClariQ test set, presented in Table 6. We use a two-tailed Welch’s t-test with a significance level of $\alpha = 0.05$ [8]. The null hypothesis ($H_0$) states that there is no statistically significant difference between the two models for a given metric.

During the comparison with Llama-3.2, the performance of BART is found to be significantly superior across most metrics, often with very high confidence ($p < 0.0001$). Here, the average effect size is also large (Cohen’s d = 3.089), indicating a large and meaningful difference. This confirms our initial observation that the choice of base model is an important factor in the fine-tuning method, since Llama-3.2 is significantly less adaptable to our specific task than Gemma-3 or Qwen2.5. It further shows that Llama-3.2’s architecture or pre-training is less suitable for this specific stylistic paraphrasing task. The only two metrics where no statistical significance exists are BERTScore-F1 and Self-BLEU. This suggests that although the model’s ability to achieve specific aspects required by the task is significantly worse across the rest of the models, the overall semantic alignment is lower and the diversity is statistically comparable to BART’s. This confirms that while PEFT can match the performance of full fine-tuning, its success is highly dependent on the suitability of the base model to the task.

On the other hand, our tests reveal that Gemma-3 and Qwen2.5 are consistently competitive with the fully fine-tuned BART. For both models, a statistically significant difference is only found for the BLEU metric ($p < 0.05$). For all other metrics, the null hypothesis cannot be rejected, meaning that the *performance of PEFT Gemma-3 and Qwen2.5 is statistically indistinguishable from that of the fully fine-tuned BART model*. Furthermore, the average effect size is small for both comparisons (0.260 for Gemma-3, 0.446 for Qwen2.5), confirming that even when there is a statistical difference, its practical magnitude is limited. These findings show that PEFT LoRA, when applied to well-suited base models, is a strong alternative to full fine-tuning for our specific task. The lack of a statistically significant difference across all semantic and fluency metrics except for one implies that the computational and storage benefits of PEFT LoRA are achieved with an insignificantly negative impact on generation quality. Moreover, the fact that a statistically significant difference is only found for the BLEU metric suggests that while there may be minor variations in phrasing, the core semantic quality and task performance are successfully replicated.

### Qualitative analysis

To provide a deeper understanding of the performance differences observed in our results, we conduct a qualitative analysis of the generated outputs. Table 7, Table 8, and Table 9 present a selection of examples from the ClariQ, MIMICS and USi test sets, respectively, comparing the outputs of the models with the ground truth.

We can see that our qualitative results are in line with our numerical results in Table 5. The PEFT LoRA models show consistency and accuracy, with both Gemma-3 and Qwen2.5 producing nearly identical outputs with the ground truth. Even Llama-3.2 correctly learns the stylistic transformation for this straightforward case, despite the repetition, and despite the fact that it significantly underperforms in the quantitative metrics. This again shows that the PEFT LoRA is a reliable and suitable method for the PAA-to-CQ translation task in this context.

On the other hand, prompt engineering approaches display noteworthy instability and a high failure rate in the qualitative sense. For example, Llama-3.2 with few-shot prompting completely fails to perform the task, instead providing a generic and off-topic response (“I can provide information on topics related to technology...”). Furthermore, Gemma-3, particularly with zero-shot and few-shot prompts, produces highly irrelevant outputs, often completely going off-topic and hallucinating towards the end of the generated output with snippets of foreign languages and non-ASCII characters. And when the generated utterances are somewhat related to the PAA, the model still asks unrelated clarifying questions of its own. For instance, in the ClariQ dataset, the only acceptable output is from Gemma-3 with prompting is with the instructional few-shot prompting, where the model is able to generate the desired output style, only to hallucinate at the end with “iexplore?”.

Another common trend we observe is that models that generate somewhat relevant content to the original task still change the intent of the CQ, re-inventing the context of the conversation. Some examples of this are “Does anyone else see any bright birds flying around here?” from Llama-3.2 with instructional few-shot prompting for ClariQ, and “Can you tell me some tips on choosing a good home theater system?” from Qwen2.5 with few-shot prompting for USi. These answers are usually grammatically correct, and not extremely off-topic from the PAA, but are semantically completely different from the expected output format of the task.

Finally, a notable exception to the failure of prompt engineering is the Qwen2.5 with the few-shot instructional prompting, which successfully generates a perfect paraphrase from PAA-to-CQ. This strongly supports one of the main findings of our study: while prompt engineering can succeed with the right combination of an instruction-following model and a suitable prompt, fine-tuning provides a far more reliable, consistent, and generalizable method for creating CQs from PAA’s, even when computationally efficient techniques are selected over full fine-tuning.

### Limitations

While this study provides an extensive comparison between fine-tuning and prompt engineering for the PAA-to-CQ task, it is important to acknowledge its limitations.

One significant disadvantage we have compared to our initial work is that we lack the computational resources to fully fine-tune the models we use. That, combined with the fact that we aim at experimenting with the current state-of-the-art large language models, makes the performance of our models slightly lower in some situations, even though we have observed this difference to be statistically insignificant. However, one benefit of this is that our findings demonstrate that the task can be performed with less computationally demanding setups.

Moreover, our study intentionally focuses on lightweight, open-source models, in the 1–1.5B parameter range. This suggests that our results might sometimes not generalize to larger-scale models. The large gap in performance between fine-tuning and prompt engineering might narrow with larger models with more powerful in-context learning capabilities. While we ensure that our experiments are accessible and reproducible, it is important to note that our conclusions about model and method performance should be taken within the context of small-scale, open-source models.

Finally, our study is subject to considerations inherent in LLM-based research. The first is the possibility of existing biases in the models’ pre-training data to influence the generated outputs, which could affect how different topics are rephrased. The second is the fact that our methodology relies heavily on the availability of Google’s “People Also Ask” (PAA) feature as a source of candidate questions, as well as the quality of the suggestions made by the PAA feature. This may not be feasible for scenarios there are no PAAs, or where the PAA questions themselves are of low quality or irrelevant to the user’s initial query.

## Conclusions and future work

In this work, we conduct an extensive investigation into generating clarifying questions (CQs) for conversational search by transforming questions from Google’s “People Also Ask” (PAA) feature. We compare two machine learning paradigms: parameter-efficient fine-tuning (PEFT) with LoRA, and various prompt engineering strategies, i.e. zero-shot, few-shot, and instructional few-shot, across three state-of-the-art, lightweight, open-source language models. Our findings show that fine-tuning significantly outperforms prompt engineering for this task, achieving high lexical and semantic similarity to human-generated ground truths and generalizing well across three distinct test sets.

Furthermore, we show that the performance of our PEFT LoRA models is statistically indistinguishable from that of a fully fine-tuned legacy model from previous work [8], while offering significant computational and resource efficiency. On the other hand, prompt engineering methods struggle to match similar levels of consistency and accuracy, and often produce off-topic or hallucinating outputs. We observe that for the PAA-to-CQ translation task, investing in a small, high-quality dataset for fine-tuning is a more reliable and effective strategy than relying on in-context learning alone, even when the fine-tuning is done in a small scale.

The impact of this work includes but is not limited to observations regarding the selection and usage of LLMs for this task. We see that for smaller models, prompting strategies struggle with producing consistent results and are prone to creating hallucinations. Furthermore, the variation in performance between the three models we use suggests that base-model architecture and pre-training remain critical factors, and the base model should be matched carefully to the specific linguistic requirements of the task.

Regarding the broader field of CIR, this study demonstrates the possibility of shifting from static to dynamic question banks for creating CQs. By using PAAs for our reference points for the CQs, we show that CIR systems can generate questions that are not only conversationally appropriate but also are backed by real user behaviour. This way, we ensure that the questions asked by the system are timely, accurate, and aligned with real-world information needs. To support further research in this matter, we introduce two new, human generated test sets. These benchmarks, along with the use of a diverse set of metrics should provide a baseline for CQ generation with PAAs.

Building on the findings of our work, several possibilities for future research appear. An extension is to replicate these experiments with larger language models, in the 7B to 70B parameter range. We predict that the performance gap between fine-tuning and prompt engineering will narrow as the model size grows. While our prompting strategies show promise for instructional few-shot prompting, more advanced techniques such as Chain-of-Thought [41] can be explored to guide small models to follow certain stylistic constraints with higher success rate.

Furthermore, our work assumes a relevant PAA question is already selected. Future work can explore a more comprehensive system, which would first rank a set of candidate PAA questions based on pre-defined metrics for usefulness, and then generate the CQ from the top-ranked candidate. Other user-generated content could also be leveraged, such as forum discussions to generate CQs, comparing them against PAAs.

Finally, while automatic metrics provide a strong quantitative baseline, future work could introduce human evaluation to assess the quality of generated CQs in a live conversational search environment, measuring the impact on task success and user satisfaction. These directions can help further refine the generation of high-quality clarifying questions, moving closer to more natural and effective conversational search systems.

## Footnotes

1 See: https://www.google.com/search/howsearchworks/.

2 See: https://support.google.com/websearch/answer/9351707.

3 See: https://github.com/huggingface/peft.

4 Data and code will be made available upon request.

5 https://github.com/huggingface/peft.

6 https://huggingface.co/docs/transformers/en/main_classes/trainer.
